# Supplementary material for: Pulmonary Function in Post-Infectious Bronchiolitis Obliterans in Children: A Systematic Review and Meta-Analysis
Source: Pathogens. 2022 Dec 14;11(12):1538. doi: 10.3390/pathogens11121538 (PMC9780806; doi:10.3390/pathogens11121538)
Supplement: Supplementary file 1 [file pathogens-11-01538-s001.zip › pathogens-2040418-supplementary.pdf]

Supplementary Table S1. Summary of levels of key pulmonary function and lung volume parameters in children with PIBO

Variables	Number of studies	Normal reference value	TE			Q statistics			I^2			*P* for Begg's rank test
			Estimate	Lower	Upper	Estimate	DF	*P* value	Estimate	Lower	Upper	
Pulmonary function parameters												
FEV_1 % predicted	10	80%	51.367	44.227	58.507	155.432	9	<0.001	0.942	0.913	0.962	0.245
FEV_1 z score	5	0	-2.576	-4.236	-0.917	364.656	4	<0.001	0.989	0.984	0.992	0.624
FEV_1 (L)	2		1.403	0.375	2.431	17.766	1	<0.001	0.944	0.825	0.982	
FVC % predicted	10	80%	68.387	64.443	72.331	41.6185	9	0.006	0.7838	0.606	0.881	0.531
FVC z-score	5	0	-1.860	-3.213	-0.507	212.623	4	<0.001	0.981	0.971	0.988	0.142
FVC (L)	2		2.231	1.163	3.298	17.600	1	<0.001	0.943	0.822	0.982	
FEV_1/FVC (%)	8	70%	68.817	62.184	75.449	129.978	7	<0.001	0.946	0.915	0.966	0.138
FEV_1/FVC z-score	4	0	-2.044	-2.547	-1.540	30.641	3	<0.001	0.902	0.779	0.957	0.174
$FEF_{25\text{-}75\%}$, % predicted	8	65%	26.489	19.336	33.642	221.978	7	<0.001	0.969	0.954	0.979	0.458
$FEF_{25\text{-}75\%}$ z-score	4	0	-2.016	-3.619	-0.412	248.244	3	<0.001	0.988	0.981	0.992	0.497
$FEF_{25\text{-}75\%}$, L	1		0.500	0.412	0.588							
PEF, % predicted	2		64.437	43.577	85.298	17.736	1	<0.001	0.944	0.824	0.982	
PEF (L)	1		0.500	0.412	0.588							
Change in FEV_1 after bronchodilator (%)	3		6.063	4.890	7.236	19.539	2	0.005	0.898	0.725	0.962	0.117
Lung volume parameters												
TLC % predicted	6		121.304	113.276	129.332	37.710	5	<0.001	0.867	0.734	0.934	0.348
TLC, L	3		2.874	0.377	5.371	301.737	2	0.001	0.908	0.668	0.974	0.117
RV, %	8		287.782	244.455	331.110	151.699	7	<0.001	0.954	0.929	0.970	0.458
RV, L	2		0.191	-3.277	3.660	0.001	1	0.983				

RV/TLC, %	5		85.905	65.083	106.727	789.533	4	<0.001	0.995	0.993	0.996	0.142
RV/TLC, L	1		59.100	55.419	62.781							
Other												
DLco, % predicted	2	75%	64.921	45.571	84.272	29.828	1	<0.001	0.967	0.910	0.988	

CI, confidence interval; DF, degree of freedom; DL_{CO}, diffusion capacity for carbon monoxide; $FEF_{25\text{-}75\%}$, forced expiratory flow between 25% and 75% of vial capacity; FEV_1, forced expiratory volume in the first second; FVC, forced vital capacity; L, liter; PEF, peak expiratory flow; PIBO, post-infectious bronchiolitis obliterans; RV, residual volume; SD, standard deviation; TE, treatment effect; TLC, total lung capacity.
